# Performance Enhancement of Consumer-Grade MEMS Sensors through Geometrical Redundancy

**DOI:** 10.3390/s21144851

**Published:** 2021-07-16

**Authors:** Giorgio de Alteriis, Domenico Accardo, Claudia Conte, Rosario Schiano Lo Moriello

**Affiliations:** 1 Department of Industrial Engineering, University of Naples Federico II, Piazzale Tecchio 80, 80125 Naples, Italy; domenico.accardo@unina.it (D.A.); rschiano@unina.it (R.S.L.M.); 2 Department of Management Information and Production Engineering, University of Bergamo, 24044 Bergamo, Italy; claudia.conte2@unina.it

**Keywords:** accelerometer, Allan variance, GNSS/INS, gyroscope, inertial measurement unit, inertial navigation systems, integrated navigation, MEMS, sensor redundancy, unmanned aircraft systems

## Abstract

The paper deals with performance enhancement of low-cost, consumer-grade inertial sensors realized by means of Micro Electro-Mechanical Systems (MEMS) technology. Focusing their attention on the reduction of bias instability and random walk-driven drift of cost-effective MEMS accelerometers and gyroscopes, the authors hereinafter propose a suitable method, based on a redundant configuration and complemented with a proper measurement procedure, to improve the performance of low-cost, consumer-grade MEMS sensors. The performance of the method is assessed by means of an adequate prototype and compared with that assured by a commercial, expensive, tactical-grade MEMS inertial measurement unit, taken as reference. Obtained results highlight the promising reliability and efficacy of the method in estimating position, velocity, and attitude of vehicles; in particular, bias instability and random walk reduction greater than 25% is, in fact, experienced. Moreover, differences as low as 0.025 rad and 0.89 m are obtained when comparing position and attitude estimates provided by the prototype and those granted by the tactical-grade MEMS IMU.

## 1. Introduction

Micro Electro-Mechanical Sensors for inertial measurements, such as gyros and accelerometers, are of high interest in the development of modern vehicles. Compact, lightweight, reliable, and affordable solutions are needed to develop autonomous transport platforms, such as Unmanned Aircraft Systems, Unmanned Ground Vehicles, and Autonomous Underwater Vehicles [1,2,3]. New needs are also determined for platforms with human passengers on-board, such as Urban Air Mobility aircraft, Autonomous Cars, and Autonomous Ships [4,5,6]. Typically, MEMS inertial sensors are included in integrated navigation packages with one or more aiding systems to control the intrinsic inertial measurement drift by means of sensor data fusion [7,8]. Improved technical solutions are made available by fast-developing innovation frameworks, such as Internet of Things IoT and Industry Automation [9]. These solutions include a large number of electronic components used for indoor and outdoor navigation that can be used as aiding sensors, such as Radio-Frequency positioning systems, air data sensors, logs, altimeters, depth sensors, echo sounders, ultrasonic sensors, and several types of imaging cameras. They are all compact electronic components that can be integrated with MEMS inertial sensors to realize small and lightweight units that can be installed on advanced transport platforms.

MEMS sensors still have some significant limitations. Actually, gyros are more critical than accelerometers since their overall accuracy is poor with respect to advanced solutions, such as optical and mechanical ones. In particular, the bias instability can be very large. Consumer-grade MEMS gyros can be affected by a bias instability in the order of 50 degrees per hour or more [9], while advanced sensors can reach tactical grade levels with a bias instability in the order of 10^−1^ degrees per hour [10]. Tactical grade is defined as the condition when the gyro can measure the Earth rate, i.e., 15 degrees per hour; this term allows for performing autonomous initialization of heading by gyrocompassing [11]. The bias instability of gyros is the most important error term that defines the overall performance of an Inertial Measurement Unit. It can be divided into two contributions, such as bias thermal drift and bias drift [5]. The first contribution can be reduced by means of calibration [12]; the negative effects of the second one are controlled by means of sensor data fusion. Typically, it is considered as an additional state parameter with respect to navigation errors to form Augmented-state Extended Kalman Filters [5]. In this framework, MEMS gyro bias drift shall be reduced as much as possible to keep the overall error within acceptable performance levels. If no single sensor is available with satisfactory bias performance, the use of redundancy is an option to realize proper solutions. It is worth noting that using MEMS gyros instead of optical ones results in a very large gain in terms of space, weight, power consumption, and costs. For this reason, part of the gain can be sacrificed to increase the number of sensors, thus also increasing the accuracy of navigation state estimates by integrating the measurements of redundant gyros and accelerometers. Redundancy can be considered as a special form of sensor data fusion, where the integrated measurement comes all from sensors of the same type. The benefits of redundancy are not just related to accuracy. Redundancy can also be effective in increasing the overall reliability, integrity, and robustness of the system [5]. For instance, redundant sensor configurations provide graceful degradation of accuracy in case of single-sensor failure [13].

The availability of rapid-prototyping electronic boards, such as those provided by STMicroelectronics™ [14], permits us to perform a fast arrangement of prototypical redundant units to test the effectiveness of redundant solutions for advanced transport systems applications. With the aim of improving the measurements obtained by consumer-grade MEMS inertial sensors, the paper proposes an innovative method to integrate redundant inertial measurements. A prototypical unit was made up of six sensor boards, each comprising MEMS triaxial accelerometer and triaxial gyroscope, in a redundant configuration. The unit has been experimentally tested with indoor and outdoor trials to demonstrate the attainable levels of accuracy. The testing plan includes an Allan variance test of redundant sensors and field test of integrated navigation with Global Navigation Satellite System (GNSS) receivers. The first has been performed to verify the overall bias performance of redundant configuration. The second has been realized to assess if the output of integrated navigation is compliant with the requirements for modern autonomous transport systems. At the end of the paper, test results are discussed to show the reached quasi-tactical grade capability.

## 2. Theoretical Background

To overcome known limitations affecting the performance of Inertial Navigation Systems (INS) based on consumer-grade MEMS sensors, a redundant configuration of cost-effective accelerometers and gyros placed on the faces of regular polyhedron has been investigated. Thanks to the offered opportunity of averaging acceleration and angular rate values coming from different sensors, the considered geometric configuration allows a preliminary self-calibration and/or compensation of typical inertial sensors uncertainty sources (such as bias instability and random walk-driven drift, in the following referred also to as bias drift for the sake of brevity), thus reducing the overall uncertainty in attitude, position and speed measurements [15]. To better appreciate the proposed method, some fundamental theoretical notes about the main topics are given in the following.

### 2.1. Advantages of Redundant Configuration

Thanks to a reasonable assumption of lack of correlation, exploiting a redundant configuration allows to suitably reduce the typical errors (such as bias instability, bias drift, scale factor drift [12]) with respect to that affecting each single inertial unit. To this aim, let us consider a geometrical distribution involving *N* sensors whose sensing axes are characterized by an invariable orientation with respect to the redundant Inertial Measurement Unit (IMU) reference system. Let $x_{k}$ be the generic quantity (either acceleration or angular rate) measured by the *i*-th inertial sensor; according to [12,16] the measured values can be expressed as:

(1) $$x_{k}=x_{r}+SF_{i}x_{r}+b_{k}+\varepsilon_{k}+Nx_{r}$$

where $SF_{i}$ represents the scale factor, $x_{r}$ is the actual measurand, b stands for the bias, ε is the random noise, and, finally, N is the skew-symmetric matrix that contains the non-orthogonalities. Even though both scale factor and bias affect sensor measures, in the following the attention will be focused on bias; similar considerations hold for the scale factor. Moreover, let $\sigma_{i}$ be the standard deviation of the sensor bias associated with the *i*-th axis; even though different sensors would be characterized by different values of standard deviation, this value can be considered equal for all the sensors in the following, without loss of generality. Acceleration and/or angular rate values coming from each sensor axis can be combined by averaging their projections on each IMU axis. This way, the standard deviation $\sigma_{j,r}$ of the bias affecting the redundant configuration can straightforwardly be evaluated according to

(2) $$\sigma_{j,r}=\frac{\sigma_{i}}{\sqrt{\sum_{i=1}^{N}\cos^{2}\vartheta_{i,j}}}$$

where $\vartheta_{i,j}$ stands for the angle between the *i*-th sensor and *j*-th IMU axis, respectively.

When the *i*-th sensing axis of each sensor is parallel to *j*-th IMU axis, Equation (2) turns into

(3) $$\;\sigma_{j,r}=\frac{\sigma_{i}}{\sqrt{N}}$$

i.e., the standard deviation corresponding to the arithmetic mean of *N* values that represent a theoretical value of the standard deviation [17,18].

In order to assure a three-dimensional uniform error along any direction of each IMU axis, inertial sensors should be placed on the faces of a regular polyhedron; if this is the case, directions orthogonal to each face are characterized by spatial uniform distribution.

The coefficient $\beta_{imp}$ (i.e., the fraction denominator of Equation (2)), defined according to

(4) $$\beta_{imp}={\left(\sqrt{\overset{N}{\underset{i=1}{\sum }}\cos^{2}\vartheta_{i,j}}\right)}^{-1}$$

allows to appreciate reduction of bias standard deviation associated with the availability of independent redundant measures of acceleration and angular rate.

As reported in [15], it is worth noting that the improvement $\beta_{imp}$ is independent from the specific chosen polyhedron in terms of shape and number of faces; on the contrary, it only depends on the number *N* of exploited sensors. Moreover, values given in [15] refer to ideally oriented sensing axes; as a matter of practice, misalignments among sensors can occur, even though their magnitude can be made negligible by means of a proper calibration procedure.

### 2.2. Sensing Axes Alignment

In order to correctly combine data acquired from different sensors, their spatial orientation must be referred to a common reference frame. Different approaches (direction cosines, Euler rotation angles or quaternions) can be exploited to transform a vector from one coordinate system into another. All rotations involve a transformation matrix or Direction Cosine Matrix (DCM), usually referred to as $C_{b_{i}}^{b_{r}}$, where *b_i_* is the starting reference of the *i*-th sensor body frame, while *b_r_* is the body reference frame of destination.

The transformation matrixes are defined by a sequence of three rotations comprising Euler’s angles ϕ (roll), θ (pitch), ψ (heading) [19]. The Euler’s angles are three angles introduced by Euler to describe the orientation of a rigid body. Indeed, the relative orientation between two any Cartesian frames can be described by Euler’s angles. The advantage of Euler’s angles consists of the fact that they allow us to easily understand the orientation of a body with respect to a certain reference system. However, the main disadvantage is their propensity to singularity. Therefore, the most effective way to parametrize the transformation matrix is to use the quaternion method [5].

The quaternion representation is based on Euler’s rotational theorem, which states that the relative orientation of two coordinate systems can be described by only one rotation about a fixed axis [20]. Therefore, the quaternion is defined by a rotational axis and a rotation angle [20]

(5) $$q={\left[q_{0}\;q_{x}\;q_{y}\;q_{z}\right]}^{T}=\left[\begin{array}{c} cos\left(\frac{\Theta }{2}\right)\\ ||\vec{e}||\cdot \sin\left(\frac{\Theta }{2}\right) \end{array}\right]$$

where $\left||\vec{e}|\right|$ is the normalized rotational axis and Θ is the rotation angle.

The optimal transformation matrix of the sensing axes of each sensor can be determined by minimizing the loss function:

(6) $$J\left(A\right)=\overset{n}{\underset{i=1}{\sum }}w_{i}\;{\left|{\hat{u}}_{B}^{i}-A{\hat{u}}_{R}^{i}\right|}^{2}$$

where *A* is the transformation matrix, ${\hat{u}}_{B}^{i}$ is the vector measurements in the body system, $w_{i}$ is the weight of *i*-th ${\hat{u}}_{B}^{i}$ and ${\hat{u}}_{R}^{i}$ is the vector in the reference coordinate system.

The loss function could be rewritten in terms of quaternion using an algorithm based on Wahba’s [20] method:

(7) $$J^{\prime }\left(q\right)=q^{T}Kq$$

where the matrix *K* is defined as follows:

(8) $$K=\left(\begin{array}{cc} S-1\sigma & Z\\ Z^{T} & \sigma \end{array}\right)$$

Matrix *S*, scalar σ and the vector *Z* are given by:

(9) $$B=WV^{T}$$

(10) $$S=B^{T}+B$$

(11) $$Z={\left(B_{23}-B_{32},\;B_{31}-B_{13},B_{12}-B_{21}\right)}^{T}$$

(12) σ=tr(B)

*W* and *V* are matrices having as entries the normalized components of frame versors is in the body and reference coordinates system, respectively:

(13) $$W_{i}=\sqrt{w_{i}}{\hat{u}}_{B}^{i};{\;V}_{i}=\sqrt{w_{i}}{\hat{u}}_{R}^{i}$$

The result is an eigenvectors problem; in particular, it can be demonstrated [20], the quaternion associated with the optimal rotation is an eigenvector of matrix K. Therefore, the loss function in Equation (7) is a maximum if the eigenvector corresponding to the largest eigenvalue is chosen [20].

Finally, the formula described in [20] was used to obtain the corresponding optimal transformation matrix.

### 2.3. Kalman Filter

The estimate of a system state can be improved by fusing noisy data obtained from different types of sensors. A typical example is the Kalman Filter (KF), extensively described in [20], and usually applied in the linear dynamics model. The main groups of equations were “Time Update Equations” and “Measurements Update Equations”; the filter operates according to a prediction/correction model. In particular, the first set of equations corresponds to the state prediction while the second one allows to correct state through available measures [21].

Navigation applications are characterized by non-linear dynamic models; this way, a linearization approach is applied to the KF to obtain the so-called Extended Kalman Filter (EKF) [22]. Assuming that noise can be modeled as Gaussian white noise, the EKF provides a suboptimal technique for non-linear model and estimates system state by means of a least square error approach. A description of the different data fusion techniques is presented in [23,24,25,26].

### 2.4. Allan Variance

The dependence of the state estimates quality on parameters defining noise conditions of sensors outputs is known to be one of the most important issues associated with Kalman filtering. To suitably determining their values, different approaches can be pursued; stemming from their past experience [27,28], the authors exploited the Allan variance to gain the values of interest.

Allan variance was first proposed to overcome problems associated with standard deviation evaluation on an increasing sequence of acquired data. Even though it was originally used for oscillator frequency applications, Allan variance can be exploited to highlight and discriminate noise terms added to the signal of interest also in inertial sensors [16]. Allan variance operates with success in the assumptions that (i) considered signal remains constant and flat during the measurements and (ii) noise average should be zero for long-term acquisitions.

From an operating point of view, the Allan variance for a specific cluster time τ is defined as one half of the time average of the squares of the differences between sensor outputs separated by τ

(14) $$\sigma^{2}\left(\tau \right)=\frac{1}{2}\langle {\left(s\left(t-\tau \right)-s\left(t\right)\right)}^{2}\rangle$$

**Figure 1:**
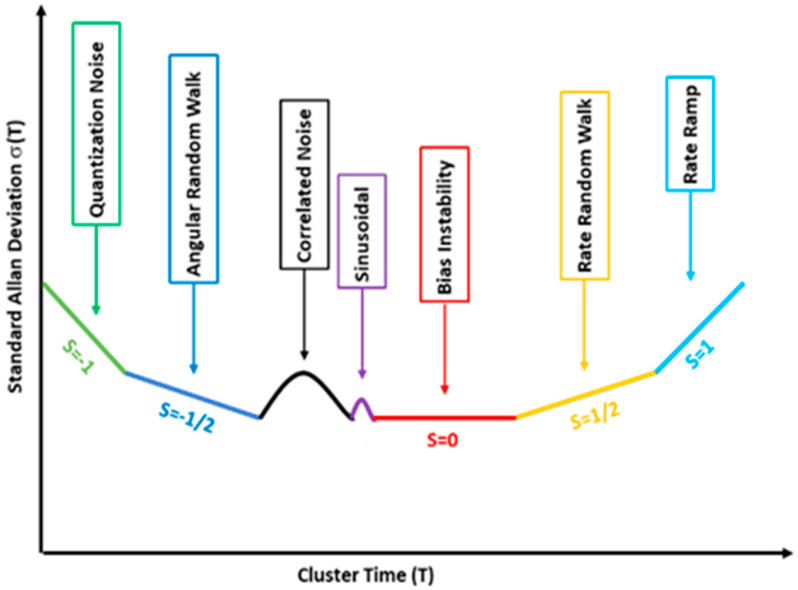
Allan deviation: characteristics parameter [16].

When inertial sensors are considered, the evolution versus τ of the Allan deviation, defined as the positive square root of Allan variance, allows us to estimate the main noise sources if a log–log scale is exploited (Figure 1). In particular, Bias Instability BI and Random Walk RW, i.e., the parameters values needed to set the entries of the noise covariance matrix, can be estimated from the curve portions characterized by slope values equal, respectively to zero and −1/2 according to:

(15) $$\sigma^{2}\left(\tau \right)=\frac{2BI^{2}\ln2}{\pi }$$

(16) $$\sigma^{2}\left(\tau \right)=\frac{RW^{2}}{\tau }$$

## 3. Proposed Method

**Figure 2:**
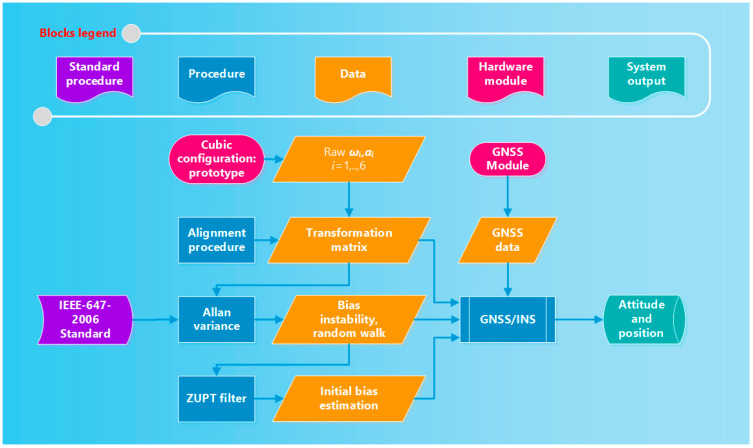
Flow chart of the measurement procedure.

To overcome the above-mentioned limitations, the exploitation of a redundant configuration of low-cost MEMS inertial sensors along with a suitable digital signal processing strategy is proposed to gain an accurate estimate of position, velocity and attitude. For the sake of clarity, the operating steps of the proposed method are shown in Figure 2. The blue blocks represent the steps of the adopted procedure, while orange blocks were the obtained data; hardware modules are indicated through a red block, while the green block stands for the output of the system. Offline operations (alignment procedure and Allan variance estimation) are once carried out when the redundant IMU is assembled, while inline processing (Zero Velocity Update (ZUPT), and GNSS integration) is real-time performed to estimate navigation parameters.

### 3.1. Alignment Procedure

According to what is stated in [29], regular polyhedra can be generated only up to twenty faces (icosahedron), in terms of feasible solutions. Among all mentioned, the authors have chosen the cubic one due to its straightforward configuration to be realized as a prototype and, mainly, the favorable condition of having a theoretical value of $\beta_{imp}$ equal to 0.41 in the presence of ideal parallelism among the same sensing axis of each sensor.

**Figure 3:**
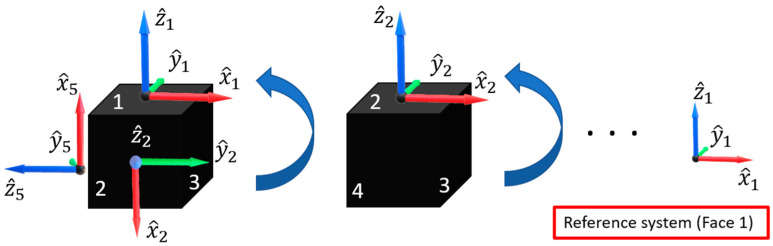
Operations of sensing axes alignment procedure.

This way, measures associated with the body frame of each inertial sensor must be reported into a single reference frame (Figure 3). A suitable alignment procedure, based on the Wahba method [30] was thus implemented to gain the rotation matrices capable of aligning the sensing axes of the accelerometers and gyros of each sensor to those of the common reference frame, arbitrarily chosen as sensors installed on face 1 of the cube. It is worth nothing that the considered procedure can align the sensing axes of each inertial sensor to the corresponding ones of the reference frame. This way, possible misalignments present in the reference frame and due to non-perpendicularity of the sensor sensitive axes and non-perpendicularity of the sensor sensitive axes with respect to the Printed Circuit Board (PCB) still remains and are, partially, shared by the realigned axes upon the procedure.

The alignment procedure must be carried out once at redundant IMU realization or when relative sensors orientation should change due to accidental modifications.

The procedure consists of the following steps:

1. The body frame of the inertial sensors installed on the face 1 of the cube is (arbitrarily) chosen as the reference frame the sensing axes of the other sensors are resolved to (Figure 3).
2. Six rotations are applied to the cube in such a way as each face of the cube is positioned with an orientation coincident with that original of face 1. As an example, in the second step of Figure 3, the second rotation (associated with the face 2) is reported;
3. For each rotation *i* (varying from 1 up to 6), raw data of acceleration $A_{ijk}=\left(ax_{ijk},ay_{ijk},\;az_{ijk}\right)$ are acquired from each sensor *j* (varying from 1 up to 6); the index *k* is associated with the acquired samples (varying from 1 up to *N*, *N* being the number of measurements carried out in each rotation);
4. The average acceleration vector associated with the *i*-th rotation is calculated for each sensor and the results are normalized according to
  (17) $${\hat{A}}_{ij}=\frac{mean\left(A_{ijk}\right)}{norm\left(mean\left(A_{ijk}\right)\right)}$$
  where *mean*(·) stands for the average operating on each axis and *norm*(·) represents the traditional norm operator for vectors;
5. The reference matrix *V* is arranged by means of acceleration components associated with face 1; in particular, the entries of the *i*-th row of the matrix *V* are equal to the components of the normalized acceleration of the rotation *i*
  (18) $$V=\left[{\hat{A}}_{11};\;{\hat{A}}_{21};\ldots ;{\hat{A}}_{61}\right]$$
6. As for the matrices *W_j_*, their entries can be determined in a similar way
  (19) $$W_{j}=\left[{\hat{A}}_{1j};\;{\hat{A}}_{2j};\ldots ,{\hat{A}}_{6j}\right]$$
7. According to the procedure presented in Section 2.2, a MATLAB^®^ algorithm is performed to evaluate the alignment matrices. In particular, once the matrix $K_{j}$ is calculated according to Equation (8) for each face, the optimal rotation (i.e., that capable of making the *j*-th reference frame as close as possible to the first one) is determined as a quaternion (eigenvector) corresponding to the maximum eigenvalue of the Equation (7).
8. Finally, the optimal quaternion is transformed in the related rotation matrix by means of straightforward calculation; obtained matrices are exploited to align all the measured inertial quantities to the reference frame of face 1.

Once the measurements have been resolved in a single reference and the misalignments due to the arrangement of the cubic structure have been compensated, the raw realigned measures of acceleration and angular rate are averaged for each sensing axis in order to obtain the input data for the successive navigation algorithm; as stated above, the averaging operation allows a first reduction of error source effects on the navigation performance.

### 3.2. Noise Parameter Determination

Bias affecting inertial sensors output are compensated by means of Kalman filters. A key issue is the determination of the entries of the so-called covariance matrix of the process noise, usually indicated as *Q*. Without loss of generality, *Q* can be determined as a diagonal matrix whose entries are the variance associated with bias instability and random walk of either accelerometers or gyros. Suitable values of the matrix *Q* promote a proper convergence of the Kalman filter, thus allowing to accurately estimate navigation parameters. Allan deviation approach has been exploited to set the desired values.

To this aim, measures of accelerations and angular rates provided by the redundant configuration have been acquired upon an observation interval of 72 h with a sampling period *t_c_* equal to 8 ms; this way, a whole number of samples *M* equal to 32.4 MSamples for each sensing axis has been acquired. Allan variance is evaluated for a specific cluster time *τ* = *mt_c_* from raw digitized data according to

(20) $$\sigma^{2}\left(mt_{c}\right)=\frac{1}{2{\left(mt_{c}\right)}^{2}\left(M-2m\right)}\overset{M-2m}{\underset{k=1}{\sum }}{\left(S_{k+2m}-2S_{k+m}+S_{k}\right)}^{2}$$

where *S_k_* stands for the *k*-th sample of angle or speed sequence obtained as cumulative sum of angular rate and acceleration data, respectively.

According to Equations (15) and (16), the needed values of bias instability and random walk can be evaluated by singling out the curve portions characterized by slope equal to 0 and −0.5.

### 3.3. Initial Biases Estimation

**Figure 4:**
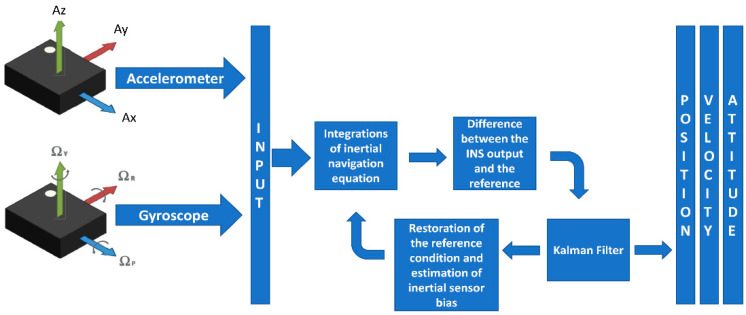
Initial bias estimation procedure.

Once the sensing axes of each sensor are determined and the noise parameters are aligned, an initial estimate of the bias is carried out by means of a ZUPT filter applied on the measurements of averaged accelerations and angular rates provided by the redundant configuration (Figure 4). To make this stage work with success, the cubic IMU is kept stationary for about two minutes and its outputs are iteratively processed through the ZUPT filter.

ZUPT filter is based on the Kalman Filter that evaluates in the prediction stage the navigation parameters (position, velocity, and attitude) obtained as an integration of acceleration and angular rate data.

Successively, the predicted position and velocity are compared in the correction stage with those unchanging of the stationary condition. Any difference between the considered values only arises because of the biases that can be thus estimated and given as output from the ZUPT.

### 3.4. Kalman Filter-Based Navigation Algorithm

Once the initial estimates of biases are determined, acceleration and angular rate data provided by the cube can be exploited to determine the navigation parameters of the redundant inertial unit. Different approaches can be adopted to update the values of the biases cyclically; in particular, an extensive review of techniques based on the integration of low-cost INS and GNSS can be found in [31]. In the realized redundant prototype, a loosely coupled integration algorithm, exploiting an Extended Kalman Filter (EKF), has been adopted as data fusion technique. In particular, INS outputs are processed by means of traditional navigation equations in order to achieve an a priori estimate of position, velocity, and attitude of the moving body; the a priori estimates are then updated and corrected each time a corresponding measures is available from the GNSS [32,33].

The first operation accounts for the initialization of the values of position and velocity, given by GNSS data. Moreover, initial attitude is estimated through leveling operations [21].

Successively, the adopted integration Kalman filter algorithm estimates the 15-dimension state vector (involving position, velocity, attitude, bias of accelerometers and bias of gyros) by iteratively operating according to two steps:

1. Prediction/integration. At this step, the inertial navigation equations are integrated. To this aim, measures of accelerations and angular rates provided by the cube are first corrected from the last available values of bias. This stage provides the so-called a priori estimates of the state vector, i.e., a state vector updated by only integrating the corrected accelerations and angular rates; possible uncompensated biases effects make the estimated navigation parameters diverge from the actual values;
2. Correction through GNSS data. When new measures of position and velocity are available from the GNSS, their values can be exploited to correct the a priori estimates of the state vector. In particular, a suitable matrix, Kalman gain, allows us to weight the confidence between integration and GNSS data and evaluate the so-called a posteriori estimate of the state vector. The higher the values of the Kalman gain, the greater the confidence and successive correction from GNSS measures with respect to the result of the integration stage. Moreover, in this step, the biases responsible for the difference between the integration and GNSS navigation parameters are estimated and given as input for the successive prediction/integration step.

## 4. Realized Prototype of Redundant IMU

The first prototype of redundant configuration has been designed in such a way as to be exploited as a data acquisition system; in particular, raw data acquired by each inertial sensor are transmitted to an external Personal Computer (PC) running the navigation algorithm.

### 4.1. Hardware Architecture

**Figure 5:**
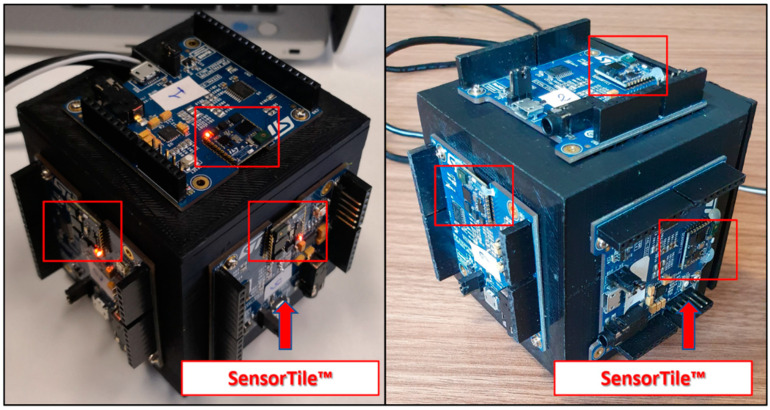
Prototype developed.

Method performance has been assessed by means of a suitable prototype of redundant IMU based on low-cost inertial sensors. The redundant inertial measurement unit has been realized by assembling six cost-effective MEMS inertial sensors on cubic support. Each couple of inertial sensors is mounted on a suitable development board, SensorTile™ from STMicroelectronics. The SensorTile^TM^ integrates on a single board a Cortex-M4-based microcontroller characterized by an operation frequency of 80 MHz and a suitable set of motion and environmental sensors. In particular, the board is equipped with a MEMS inertial module (iNemo) that includes a triaxial accelerometer and triaxial gyroscope, whose main nominal specifications are given in [34]. The microcontroller is wired-connected with the iNemo through a traditional SPI (Serial Peripherical Interface) protocol, operating as the master with a serial clock rate equal to 10 MHz. The six SensorTiles have been mounted in the redundant configuration on the faces of a 3-D printed cube made in Acrylonitrile Butadiene Styrene (usually referred to as its acronym, ABS); in particular, an ad hoc Computer Aided Design (CAD) model has been created in order to reduce shrinkage effects and obtain the minimal orthogonality error between adjacent faces (Figure 5).

Accelerations and angular rates measured by each SensorTile^TM^ are transmitted to a further microcontroller installed on a Nucleo-F303K8 development board and acting as a concentrator of acquired data. Moreover, the connection among concentrator and SensorTiles is carried out by means of the SPI protocol; in this case, the concentrator acts as master of the connection, while the serial clock period has been set equal to 100 ns. Finally, the concentrator provides a Universal Serial Bus (USB) connection with a personal computer for external data transmission and processing as well as a further SPI channel to a Micro-SD (Secure Digital) card adapter module for data saving; to this aim, a proper level shifter has been adopted to match the value of communication voltage between the concentrator and the adapter module.

The hardware architecture is complemented with a X-NUCLEO-GNSS1A1 by STMicroelectronics, i.e., GNSS expansion board based on Teseo-LIV3F module compliant with Arduino^TM^ UNO R3 connector [35]. Teseo-LIV3F is a tiny GNSS module supporting the most exploited satellite navigation constellations (Global Positioning System, GPS, BeiDou, Galileo, etc.). The GNSS module is mounted on a NucleoF401RE development board, whose microcontroller acts as an interface between the Teseo-LIV3F module and the concentrator. In particular, GNSS module is activated to provide the National Marine Electronics Association (NMEA) message $PSTMPV$ containing information about the current time, position, and speed [35]. The message is received by the microcontroller through a universal asynchronous receiver/transmitter serial (UART) protocol, operated with a baud-rate equal to 9600 bps. The microcontroller forwards the NMEA message to the concentrator by means of a further UART connection with a baud rate equal to 115.2 kbps. To suitably reduce transmission duration, the microcontroller filters out all the messages received by the GNSS module but the one containing the information required by the proposed navigation algorithm.

### 4.2. Software Architecture

With regard to the software architecture, firmware for SensorTiles, GNSS module and concentrator has been implemented through Mbed RTOS [36]; in particular, MbedCLI integrated development environment has been adopted to allow debugging facilities during the implementation stage.

To make the prototype compatible with aeronautical applications and constraints, the inertial sensor of each tile must be sampled at rate not lower than 100 Hz. On the other side, iNemo can acquire acceleration and angular rate with a lowest sampling period equal to 150 μs (as an example, for electronic image stabilization in mobile phone or camera applications). A proper tradeoff must be found between navigation and computational burden associated with the execution of the algorithm implementing the proposed method; a sampling period equal to 8 ms has thus been chosen.

As regards the measurement operations, the concentrator exploits the channel selection lines of the SPI protocol to simultaneously provide SensorTile with a trigger event each for the measurement of triaxial acceleration and triaxial angular rate. It is worth noting that an ad hoc SPI firmware has been implemented in the Tiles to reduce as low as possible the communication time usually taken by the available drivers. Moreover, configuration registers of the iNemo are set in such a way as to update inertial measures with a sample rate of 416 Hz, thus assuring new values of accelerations and angular rates for each measurement request. Finally, full-scale values of ±2 g and ±125 dps have been chosen to gain the best sensitivity.

The concentrator then polls each SensorTile^TM^ individually to obtain the measured values, already rotated to be aligned with a reference frame. To reduce the transmission interval, the output code, expressed through two-bytes signed integer representation and provided by the 16-bit analog-to-digital converter integrated into the iNemo is transmitted for each quantity of interest [33]; this way, the whole set of six inertial measurands (three accelerations and three angular rates) requires only 12 bytes to be transmitted, with a nominal time interval equal to 10 ms for each SensorTile^TM^. Received data are then forwarded to the Micro SD-card for storage and to the personal computer for navigation processing; the whole needed time is equal to about 5 ms; the remaining 3 ms are exploited by a MATLAB^TM^ application running to carry out a prediction/correction cycle of the Kalman-based navigation algorithm. As stated above, the inputs of the navigation algorithm are the averaged inertial quantities, for each sensing axes. Finally, GNSS measures are also sent to the algorithm for the correction step.

## 5. Experimental Results

Performance of proposed method along with the realized prototype has been assessed by means of a number of experimental tests with the aim of highlighting improvement brought by the redundant configuration with respect to a single couple accelerometer/gyro of sensors. In particular, the results obtained for the preliminary characterization of the prototype bias instability and drift, evaluated as described in Section 2.4 and Section 3.2, are shown. The improvement due to redundancy has then been assessed thanks to the comparison of position and attitude estimates granted by tactical-grade IMU in a real field test.

### 5.1. Reference IMU

To better appreciate achieved performance, a tactical-grade inertial sensor has been adopted as benchmark. In particular, a MEMS sensor was used, STIM300 by SensoNor™, which includes a high-performance triaxial gyroscope and triaxial accelerometer. According to what is described in the datasheet [37], the sensors assure bias instability and angular random walk equal, respectively to 0.3 °/h and 0.15 °/$\sqrt{\left(h\right)}$ for angular rate measurements; as regards accelerations, bias instability and speed random walk values are equal to 0.5 mm/s^2^ and 0.07 m/s/$\sqrt{\left(h\right)}$. It is worth noting that the inertial measures provided by the STIM300 are already compensated by an internal processing routine; nevertheless, the STIM300 output are processed by means of the integration algorithm based on Kalman filter.

### 5.2. Measurements Setup for On-Field Tests

**Figure 6:**
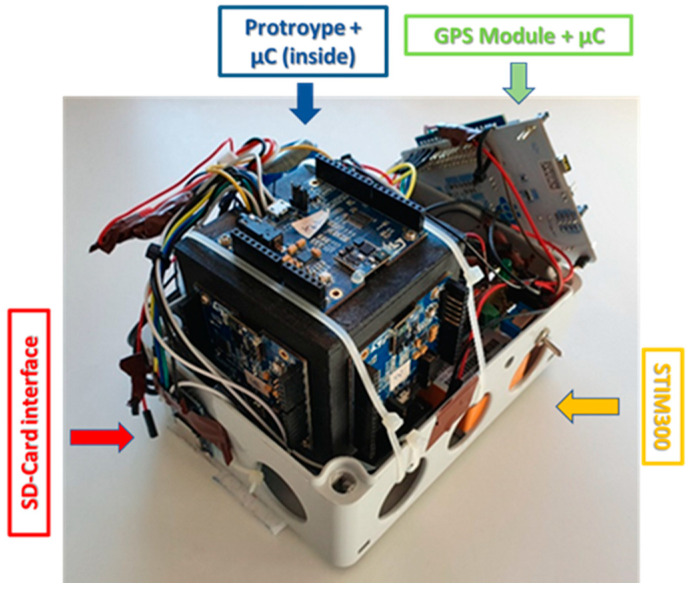
Test equipment.

The proposed method has been assessed by means of the measurement setup shown in Figure 6. The current configuration was designed and optimized for a successive exploitation on drones with a useful payload of about 0.5 kg.

The measurement setup comprised:

- 7.4 V and 2000 mAh battery for general power supply;
- DC-DC (Direct Current) step down converter circuit, to provide the adequate supply voltage for the exploited electronics;
- STIM300, used as reference IMU;
- Prototype of redundant IMU;
- GNSS module and its antenna;
- Status indicator LED;
- Two SDCard interfaces.

As for the realized prototype, a further microcontroller receives and saves on a dedicated SD-Card the reference measures provided by the STIM300. As for the initial attitude, both prototype and reference IMU have been oriented in such a way as to have the sensing axis *z* as more parallel as possible with the gravity acceleration. However, this is not a relevant constraint.

### 5.3. Preliminary Prototype Characterization

According to what was reported in Section 3, two operations must be carried out once the redundant IMU has been realized. The first one accounts for the alignment of sensing axes of each sensor on to same reference frame to suitably overcoming relative orientation and misalignment due to the prototype assembly. Obtained rotation matrices are saved on each SensorTile^TM^ that evaluates the components of measured acceleration and angular rate in the reference frame.

The residual misalignment after the realignment procedure has been assessed by evaluating the angle between the mean gravity vectors measured by reference and realigned frames. In particular, the angle α*_ij_* is defined as:

(21) $$\alpha_{ij}=mean\left(\cos^{-1}\left(\frac{g_{ij}\cdot g_{i1}}{\left||g_{ij}|\right|\left||g_{i1}|\right|}\right)\right)$$

where

- $g_{ij}$ stands for the gravity vector measured by the triaxial accelerometer mounted on the *j*-th face of the cube, aligned with face 1 after the *i*-th rotation;
- $g_{i1}$ stands for the gravity vector measured by the triaxial accelerometer mounted on the 1st (reference) face of the cube after the *i*-th rotation;
- ||·|| represents the traditional vector modulus operator.

The alignment performance is finally assessed as the average and experimental standard deviation of the 6 residual angles for each face of the redundant IMU; the corresponding results are shown in Table 1.

**Figure 7:**
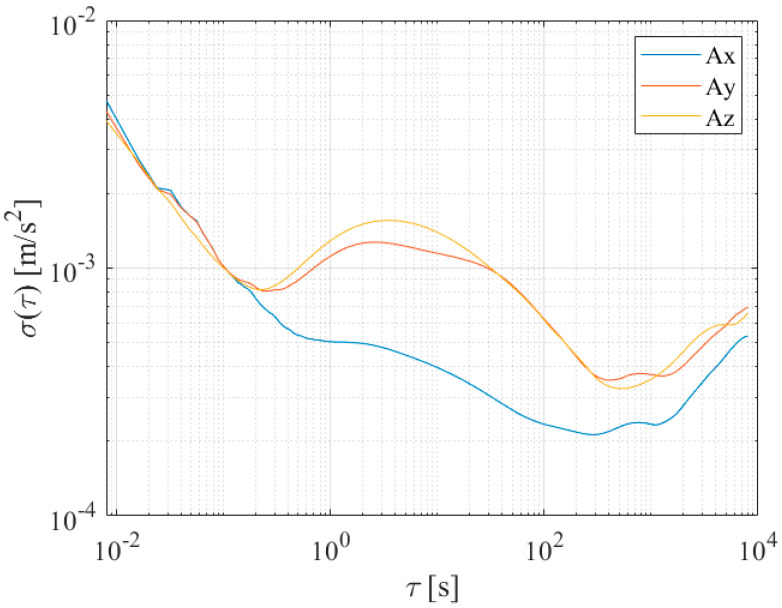
Allan deviation of accelerometers along the three sensing axes of the prototype.

**Figure 8:**
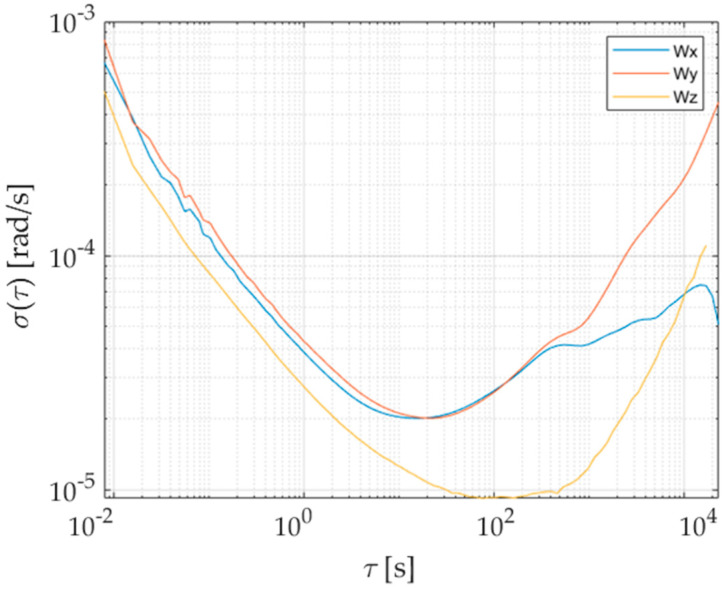
Allan deviation of gyroscopes along the three sensing axes of the prototype.

To properly estimate bias instabilities and random walks of the prototype, raw outputs of the six inertial sensors have been acquired for 48 h in a conditioned room; the measured temperature has always been within 25 ± 1 °C in order to operate as close as possible to the conditions required by the IEEE 647-2006 Standard [16]. Results of the Allan variance associated with the redundant prototype are shown in Figure 7 and Figure 8 for the accelerometer and gyroscope, respectively. The procedure described in Section 3.2 has thus been applied to gain bias instabilities (BIs) and Angular/Velocity Random Walks (ARW and VRW), whose results are shown in Table 2 and Table 3 for each sensor axis.

**Figure 9:**
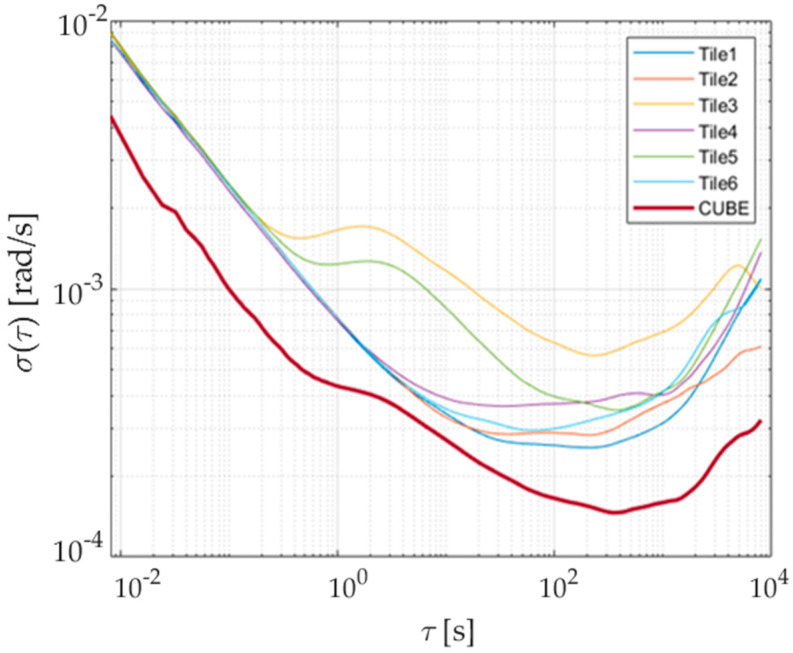
Allan deviation of accelerometers, axis *z*: single SensorTile vs. prototype.

**Figure 10:**
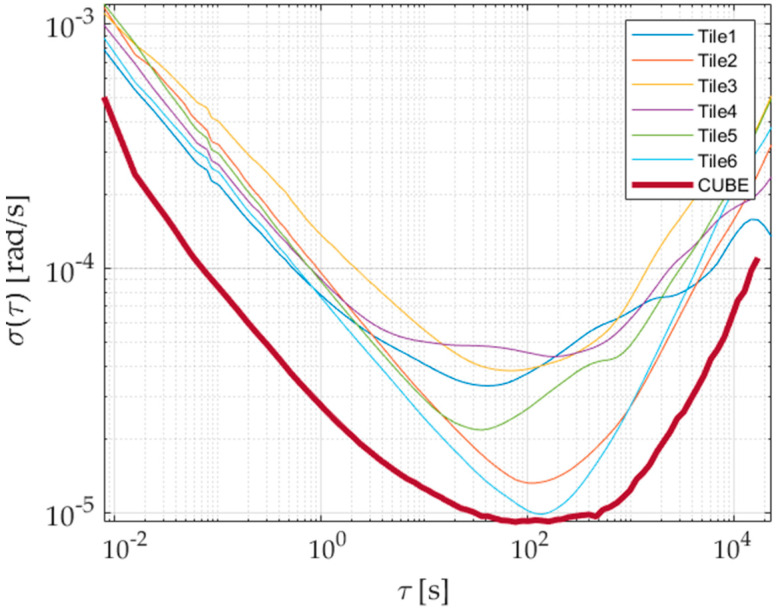
Allan deviation of gyros, axis *z*: single SensorTile vs. prototype.

To better appreciate the advantages brought by the redundant configuration, Allan variances of each individual sensor and prototype have been estimated. For the sake of clarity and simplicity, results associated with only one sensing axis for both accelerometer and gyroscope have been reported and shown Figure 9 and Figure 10; similar results and evolutions have also been experienced for the other axes. Moreover, the best bias instability and random walk experienced on each sensing axis are given in Table 2 and Table 3 for either acceleration or angular rate; performance improvement can straightforwardly be noticed. The obtained advantages can also be evidenced by comparing the prototype parameters with the estimated range of variation of the single sensor for each axis and quantity, reported in Table 4.

### 5.4. Attitude and Position

**Figure 11:**
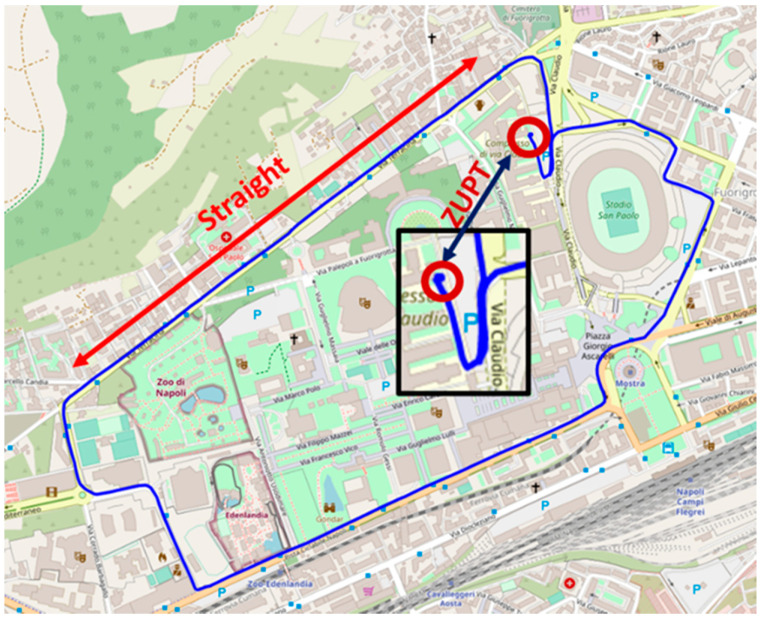
Nominal car route travelled in tests for position and attitude estimation.

Once the best entries for the process noise covariance matrix are determined, the performance of the prototype has been assessed by a real navigation condition carried out on the terrestrial vehicle. In particular, both prototype and reference IMU have experienced an urban path whose GNSS positions, acquired with a sampling period equal to 1s, are shown in Figure 11. According to the proposed method, the measurement setup has been held in stationary conditions for about 60 s to accomplish the ZUPT stage, mandated to the initial bias estimation. Successive GNSS outputs, in terms of position and velocity, have been exploited in the correction stage of the Kalman filter to finely estimate sensor parameters, thus allowing a metrological behavior of the prototype very close to the tactical-grade reference IMU.

**Figure 12:**
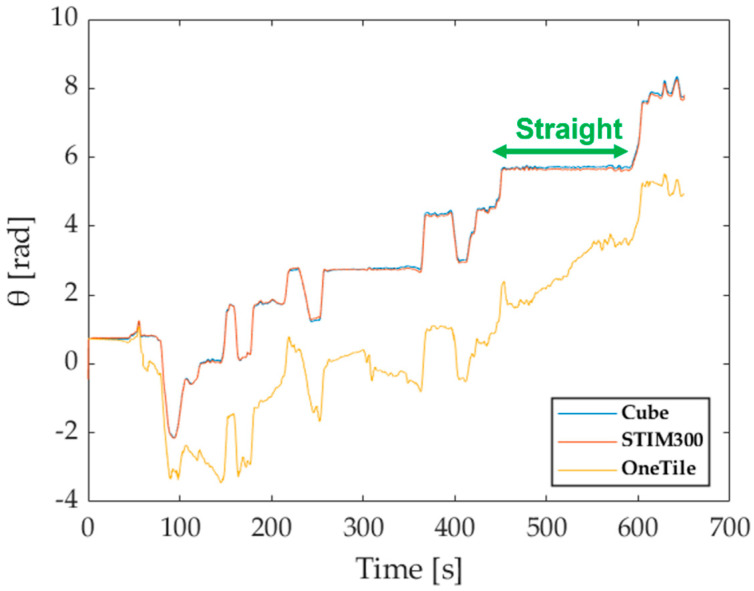
Heading angle comparison.

The first test aimed at assessing the improvement in attitude and position estimation granted by the redundant configuration; for this set of tests, all the available position and speed values of the GNSS have been considered. Heading, pitch and roll provided by both prototype and single SensorTile^TM^ have been compared with the attitude estimates granted by the STIM300; in particular, results shown in Figure 12 are associated with the heading angle, i.e., the one most difficult to be estimated due to observability issues [38,39]. Even though the prototype outputs appeared to be slower to converge with respect to the reference IMU, it is worth noting the remarkable concurrence experienced for time intervals greater than 400 s. In particular, in the route section referred to as straight in Figure 11, average differences between prototype and STIM300 angle estimates results as low as 0.025rad have been encountered. On the contrary, heading estimates obtained by means of a single SensorTile^TM^ have proven unreliable and very different from the reference values.

Moreover, with the aim of observing the benefits introduced by the redundancy, the results of the initial leveling procedure described in [5] are shown in Table 5. In particular, the acceleration measures of both single SensorTiles and prototype have been acquired upon an observation interval of 60 s in a stationary condition, and mean values as well as experimental standard deviations of estimated pitch and roll angles have been evaluated highlight the improvement granted by the proposed method.

**Figure 13:**
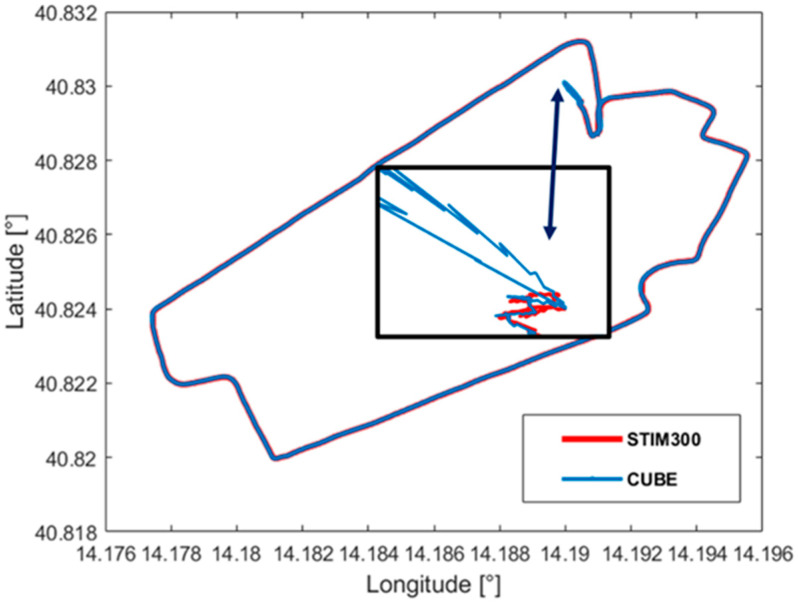
Comparison of position results provided by the proposed prototype and the reference IMU.

**Figure 14:**
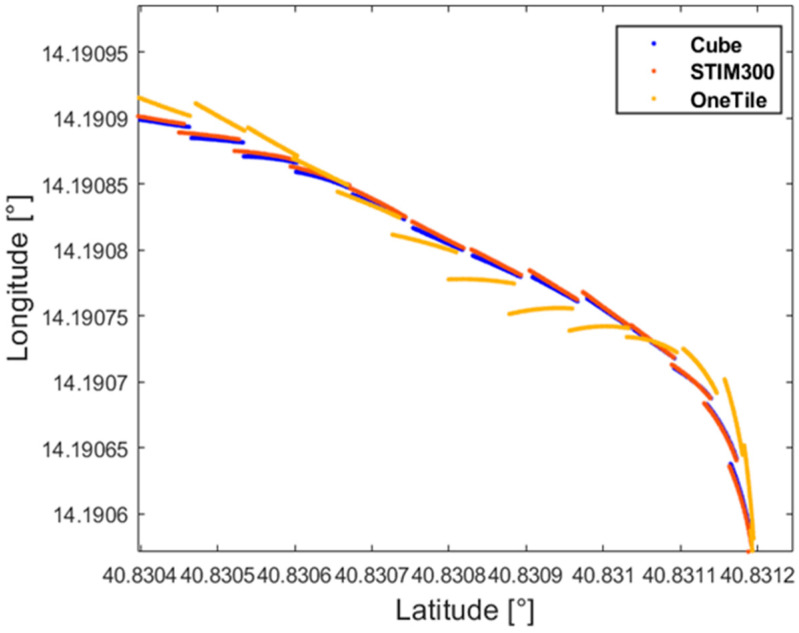
Position comparison.

Similar results have also been experienced in tests conducted for position and velocity; for the sake of brevity, the position is only presented in the following. Comparison between the positions estimated through the redundant configuration and those granted by the STIM300 is shown in Figure 13; in particular, the improvement brought by the application of the ZUPT filter can be appreciated in Figure 13. To better appreciate the performance enhancement assured by the realized prototype, a detail of the traveled path is reported in Figure 14, where the considered comparison has also involved the positions estimated by means of a single SensorTile^TM^. The discontinuities corresponded to the effect of the correction stage associated with the availability of a new GNSS output.

Further tests have been conducted to assess the robustness of the redundant configuration estimates in the presence of GNSS outages. Besides the best condition characterized by a new GNSS sample acquired each second, three other sampling periods have been taken into account (2, 5, and 10 s); moreover, to simulate typical phenomena (as an example, fading, urban canyon, and satellites loss) experienced in the urban contest and degrading the quality and availability of GNSS outputs, the set of available GNSS samples has been randomly decimated to a nominal ratio of 50%. For each sample of the traveled path, the Error Vector Magnitude (EVM) of the positions estimated by both the proposed prototype and reference IMU is calculated according to

(22) $$\mathrm{EVM}\left[k\right]=\sqrt{\begin{array}{c} {\left(x_{P}\left[k\right]-x_{R}\left[k\right]\right)}^{2}+{\left(y_{P}\left[k\right]-y_{R}\left[k\right]\right)}^{2}\\ +{\left(z_{P\left[k\right]}-z{\left[k\right]}_{R}\right)}^{2} \end{array}}$$

where *x* and *y* are the longitude and latitude estimates expressed in meter, *z* stands for the altitude, the subscripts *P* and *R* refer, respectively, to prototype and reference IMU, and k∈[1,…,N], *N* is the number of acquired samples.

**Figure 15:**
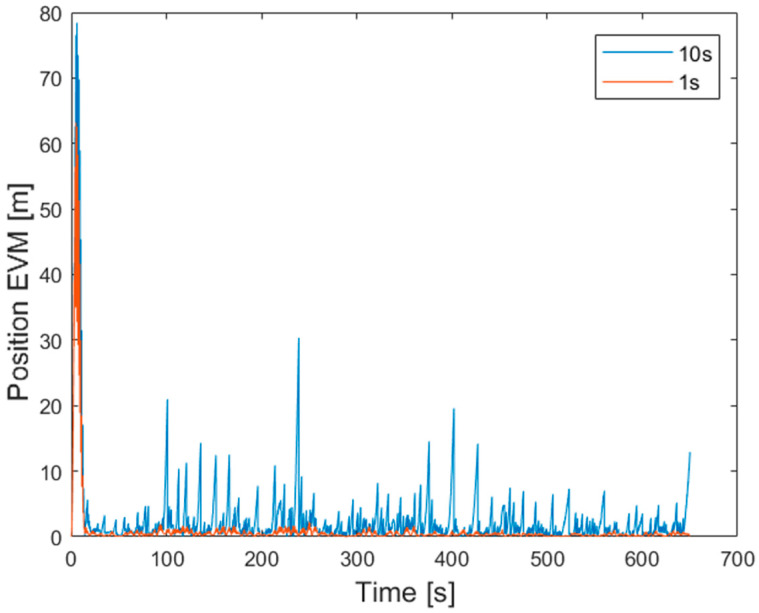
Differences of position in 1 s (red) and 10 s (blue) of outages.

As an example, the evolution of the EVM versus time is given in Figure 15 for GNSS outputs available either each second or every 10 s; despite of a limited number of spikes overcoming 20 m and associated with time instants immediately preceding the correction effect of the GNSS samples, the estimated path remarkably concurs with the STIM300 also in the presence of significant, periodic outages. The largest EVMs are encountered for samples corresponding to time instants lower than 20 s; as it can be expected, the prototype parameters are initially estimated during the ZUPT execution, thus improving the navigation performance. To better highlight, the prototype robustness to the GNSS outage, the Root Mean Square Error of the obtained EVMs (RMSE), defined as

(23) $$\mathrm{RMSE}=\sqrt{\frac{1}{N}\overset{N}{\underset{k=1}{\sum }}\mathrm{EVM}{\left[k\right]}^{2}}$$

has been exploited as a performance factor in the different GNSS outage conditions. Related results are given in Table 6, where the reliability of the proposed redundant configuration can be appreciated for outages as high as 5 s, especially in the straight path section. It is worth noting that the data portion involving the ZUPT execution has been dropped for the RMSE evaluation.

In a similar way, the robustness of attitude estimates to GNSS outages have also been carried out. In particular, the root mean square value of the differences of heading, pitch, and roll angles along the whole traveled path has been evaluated; corresponding results are given in Table 7; also, for these different operating conditions, the redundant configuration has granted attitude estimates very close to those assured by the reference IMU.

## 6. Conclusions

A method based on redundant configuration for the performance improvement in terms of bias instability and bias drift of low-cost of consumer-grade MEMS inertial measurement units has been presented hereinafter; attention has been mainly focused on bias with respect to scale factor, since the former is more relevant for this category of sensors [5]. The method has been assessed by means of a proper prototype of redundant IMU. In particular, the IMU has been implemented by arranging six SensorTile^TM^ boards by STMicroelectronics on the faces of a cube. Each SensorTile^TM^ is equipped with an inertial sensor (referred to as iNemo) consisting of 16-bit triaxial gyroscope and triaxial accelerometer exchanging raw measures with a concentrator.

According to the proposed method, the first contribution to performance enhancement is brought by (i) aligning raw measures coming from each sensor with respect to a reference frame, (ii) averaging for each sensing axis, and (iii) exploiting a GNSS-aided Kalman filter to estimate position, velocity and attitude. To further improve the performance of the redundant configuration, values of entries of the process noise covariance matrix of the Kalman filter are obtained through an Allan variance approach. According to the authors’ best knowledge, this is the first application of multiple Allan variance estimation for redundant configurations of inertial sensors. The corresponding improvement can easily be appreciated, since errors reduction with respect to the best single sensor ranged within 25% up to 80%. Moreover, initial estimates of bias for both accelerations and angular rates are achieved through the application of a ZUPT filter in a stationary condition.

Method performance has been assessed by comparing its position, velocity, and attitude estimates, obtained during an urban car test, with those granted by a commercial tactical-grade IMU, STIM300 by SensoNor. As far as heading is concerned, differences as low as 0.025 rad have been evaluated for vehicles navigation on a straight lane; corresponding values for a single-sensor navigation are much greater than 1 rad. Similar results have been achieved for pitch and roll. As for position, the performance has been expressed in terms of RMSE of the EVM of the differences of the position estimated by means of the proposed prototype and that granted by the reference. RMSE values equal to 0.89 m have been experienced for the whole car test when the update rate of the GNSS measures was equal to 1 Hz; the performance got worse in the presence of GNSS outages, reaching values of 5.25 m for an update period of the GNSS equal to 10 s.

Similar solutions have already been presented in the literature. As an example, a cubic sensor configuration has been proposed in [40] to make accurate measurements of angular velocity using a set of 12 dual-axis accelerometers. Moreover, a redundant configuration of six-axis MEMS gyroscopes as described in [41] to achieve accurate attitude estimates; to this aim, expensive navigation grade gyroscopes (with a bias instability equal to 0.02 °/h) have been exploited. On the contrary, the proposed method consists of 12 consumer-grade triaxial sensors (six accelerometers and six gyroscopes), which allows us to realize at almost the same size a complete navigation system capable of improving the overall performance of the single sensors up to the tactical grade.

The proposed method, leveraging the main features of MEMS sensors, is tailored for the implementation of an integrated navigation system for small and light unmanned vehicles, both terrestrial and aerial. However, it can be easily applied also to most performing IMU, even though the performance improvement would not be sustainable from an economic point of view. Finally, the method can be extended by considering regular polyhedrons characterized by a higher number of faces (dodecahedron and icosahedron); further improvement would be attained thanks to the face skewness and a greater number of involved sensors, to the detriment of (i) higher complexity in terms of alignment procedure and (ii) a possible reduction of the sample rate of acceleration and angular rate.

## Figures and Tables

**Table 1:** Alignment procedure accuracy.

[°]	Face 2	Face 3	Face 4	Face 5	Face 6
Mean Value	0.68	0.84	0.72	0.69	0.32
STD	0.18	0.16	0.31	0.28	0.13

**Table 2:** Gyro parameters estimated through Allan variance.

Gyroscope Allan Parameter	Prototype		Single Sensor (Best)	
	BI [°/h]	ARW [°/$\sqrt{h}$]	BI [°/h]	ARW [°/$\sqrt{h}$]
X-Axis	3.1	0.11	5.7	0.24
Y-Axis	3.1	0.11	15.7	0.36
Z-Axis	1.8	0.12	3	0.24

**Table 3:** Accelerometer parameters estimated through Allan variance.

Accelerometer Allan Parameter	Prototype		Single Sensor (Best)	
	BI [mg]	VRW $\left[m/s/\sqrt{h}\right]$	BI [mg]	VRW $\left[m/s/\sqrt{h}\right]$
X-Axis	0.02	0.01	0.05	0.04
Y-Axis	0.02	0.02	0.09	0.05
Z-Axis	0.03	0.01	0.04	0.05

**Table 4:** Variation interval, for each sensing axis, of accelerometer and gyroscope errors affecting the inertial sensors involved in the realized prototype.

Range Values [min-max]	Gyroscopes		Accelerometers	
	BI [°/h]	ARW [°/$\sqrt{h}$]	BI [mg]	VRW $\left[m/s/\sqrt{h}\right]$
X-Axis	5.7–73	0.21–0.25	0.05–0.08	0.04–0.07
Y-Axis	5.1–25	0.21–0.37	0.05–0.09	0.05–0.07
Z-Axis	3–15	0.24–0.36	0.05–0.1	0.04–0.08

**Table 5:** Initial leveling procedure comparison between prototype and single sensor.

IMU	Prototype		Single Face (Range)	
Angle [°]	Pitch	Roll	Pitch	Roll
Mean Value	0.21	1.07	−1.01 to 1.45	1.24 to 14.71
STD	0.09	0.31	0.11 to 0.45	0.2 to 0.61

**Table 6:** RMSE of position EVMs for different GNSS outages.

GPS Outages	1 s	2 s	5 s	10 s	Random
Position RMSE [m]	0.89	1.12	1.31	5.25	2.38
Position RMSE Straight [m]	0.63	0.84	1.03	3.12	1.72

**Table 7:** RMSE of position EVMs for different GNSS outages.

GPS Outages	1 s	2 s	5 s	10 s	Random
θ RMSE [rad]	0.035	0.033	0.056	0.111	0.035
θ RMSE Straight [rad]	0.037	0.034	0.023	0.051	0.025
